# Synthetic antigen-binding fragments (Fabs) against *S*. *mutans* and *S*. *sobrinus* inhibit caries formation

**DOI:** 10.1038/s41598-018-28240-0

**Published:** 2018-07-05

**Authors:** Md. Kausar Alam, Li Zheng, Ruirui Liu, Silvana Papagerakis, Petros Papagerakis, C. Ronald Geyer

**Affiliations:** 1 Department of Pathology and Laboratory Medicine, College of Medicine, University of Saskatchewan, Room 2841, Royal University Hospital, 103 Hospital Drive, Saskatoon, S7N0W8 Canada; 2 Department of Orthodontics and Pediatric Dentistry, School of Dentistry, University of Michigan, Ann Arbor, 1011N University, Ann Arbor, 48109 USA; 3 Key Laboratory of Shaanxi Province for Craniofacial Precision Medicine Research, Xi’an Jiaotong University, Xi’an, 710004 People’s Republic of China; 4 Department of Surgery, College of Medicine, Health Sciences Center, 107 Wiggins Road, Saskatoon, SK S7N 5E5 Canada; 5 College of Dentistry, Health Sciences Center, 107 Wiggins Road, Saskatoon, SK S7N 5E5 Canada

**Keywords:** Infection, Protein design, Immunization

## Abstract

*Streptococcus mutans* and *Streptococcus sobrinus* are the main causative agents of human dental caries. Current strategies for treating caries are costly and do not completely eradicate them completely. Passive immunization using nonhuman antibodies against Streptococcal surface antigens has shown success in human trials, however they often invoke immune reactions. We used phage display to generate human antigen-binding fragments (Fabs) against *S*. *mutans* and *S*. *sobrinus*. These Fabs were readily expressed in *E*. *coli* and bound to the surface *S*. *mutans* and *S*. *sobrinus*. Fabs inhibited sucrose-induced *S*. *mutans* and *S*. *sobrinus* biofilm formation *in vitro* and a combination of *S*. *mutans* and *S*. *sobrinus* Fabs prevented dental caries formation in a rat caries model. These results demonstrated that *S*. *mutans* and *S*. *sobrinus* Fabs could be used in passive immunization strategies to prevent dental caries. In the future, this strategy may be applied towards a caries therapy, whereby Fabs are topically applied to the tooth surface.

## Introduction

Dental caries is one of the most common global chronic diseases caused by the formation of biofilms on the tooth surface^1^. Gram-positive acidogenic and aciduric bacterial species, most often mutans streptococci *in humans*, have been associated with caries initiation and progression^2^. In addition to mutans streptococci (*Streptococcus mutans* and *Streptococcus sobrinus)*, other microbial species have been isolated from caries including: salivarius group streptococci (S. salivarius, S. vestibularis) and S. parasanguinis as well as lactobacilli (L. gasseri, L. johnsonii, L. casei, L. paracasei) and Veillonella species (V. atypica, V. dispar, V. parvula)^2–6^. Modern molecular and microbial culture techniques have revealed that a range of bacteria can contribute to the caries process at different stages^7^.

Dental caries are a complex biofilm-mediated disease that occurs when acidogenic/aciduric bacteria obtain a selective advantage over other members of the oral flora, disrupting the balance of the plaque biofilm and initiating the caries^8^. Mutans streptococci and lactobacilli can dominate advanced stages of caries formation where increased biofilm acidification results in the biofilm becoming less microbially diverse^9^.

The process of caries formation is modulated by complex interactions between acid-producing bacteria and host factors^9–11^. Streptococcal surface fibrillar adhesins (e.g. antigen I/II) control attachment to the surface of the tooth by adhering to salivary agglutinin. Cell-wall-associated glucosyltransferases (GTFs) produce adhesive glucans from sucrose, which cell wall-associated glucan-binding proteins (GBP) of *S*. *mutans* bind, resulting in glucan-mediated aggregation^12^. Water insoluble glucans produced by S. mutans GTFs are the most important for building the biofilm structure^13^. Other isoforms of GTFs and glucan produced by various oral streptococci contribute to biofilm formation in a synergistic manner^14^. *S*. *mutans* metabolism and acid production also contribute to the pathogenesis of dental caries^9–12^. *S*. *mutans* can metabolize a wide variety of carbohydrates, leading to the production of lactic acid. The acid diffuses into tooth enamel and dissolves the mineral underneath the tooth surface. If the mineral dissolution is not reversed, then early lesions result in caries^15^. New caries preventive therapies may be developed by inhibiting biofilm formation through suppression of mutans streptococci.

Despite efforts in promoting oral hygiene and fluoridation, approximately 35% of the global population suffers from tooth decay and cavities in permanent teeth^16^. Current strategies for treating caries are limited to removal of the diseased part of the tooth and placing a suitable restoration, which is costly and does not eradicate caries on other teeth^17,18^. As a result, dentistry is beginning to move from the surgical model for treating tooth decay (placing restorations) to identification of early carious lesions and preventing or treating them with non-surgical methods^19,20^.

Strategies for caries prevention, such as brushing, professional cleaning, antimicrobial peptides, sugar substitutes, and chemoprophylactic agents such as classical antibiotics, chlorhexidine, and triclosan are effective against dental biofilm, but their retention in the oral cavity is poor^21–24^. Passive immunization by applying mucosal vaccinations such as *S*. *mutans* and *S*. *sobrinus* antigens GTFs, antigen I/II, GBP, and virulence-associated immunomodulatory extracellular proteins (VIP) at inductive sites leads to increases in IgA secretion and can be effective in preventing caries formation^18,21^. Many other vaccine immunogens such as synthetic peptides, DNA-based active vaccines, and mucosal adjuvants have been successful in animal models^25–28^. The murine monoclonal antibody Guy’s 13^29^, which specifically recognizes the SAI/II protein of *S*. *mutans* and *S*. *sobrinus*, has been used to prevent caries formation in non-human primates and in human clinical trials^30^. A humanized scFv based on Guy’s 13 antibody is capable of aggregating *S*. *mutans in vitro*^31^. To date, no vaccines have been brought to market, mainly due to the difficulty in inducing and maintaining protective antibody in oral fluids^18^.

There has been a growing interest in using antibody fragments in passive immunization strategies to treat caries^32^. Advantages of using antibody fragments are their low production costs and enhanced tissue penetration. We used phage display to generate Fabs against *S*. *mutans* and *S*. *sobrinus*. By selecting the Fab-phage library against live bacteria, we were able to generate Fabs against antigens presented in their native context in an unbiased manner. Using this strategy, we obtained Fabs that bound to *S*. *mutans* and *S*. *sobrinus*, blocked biofilm formation *in vitro*, and prevented dental caries formation in rat caries model.

## Results and Discussion

### Selection of antigen-binding fragments (Fabs) against *S*. *mutans* and *S*. *sobrinus*

**Figure 1:**
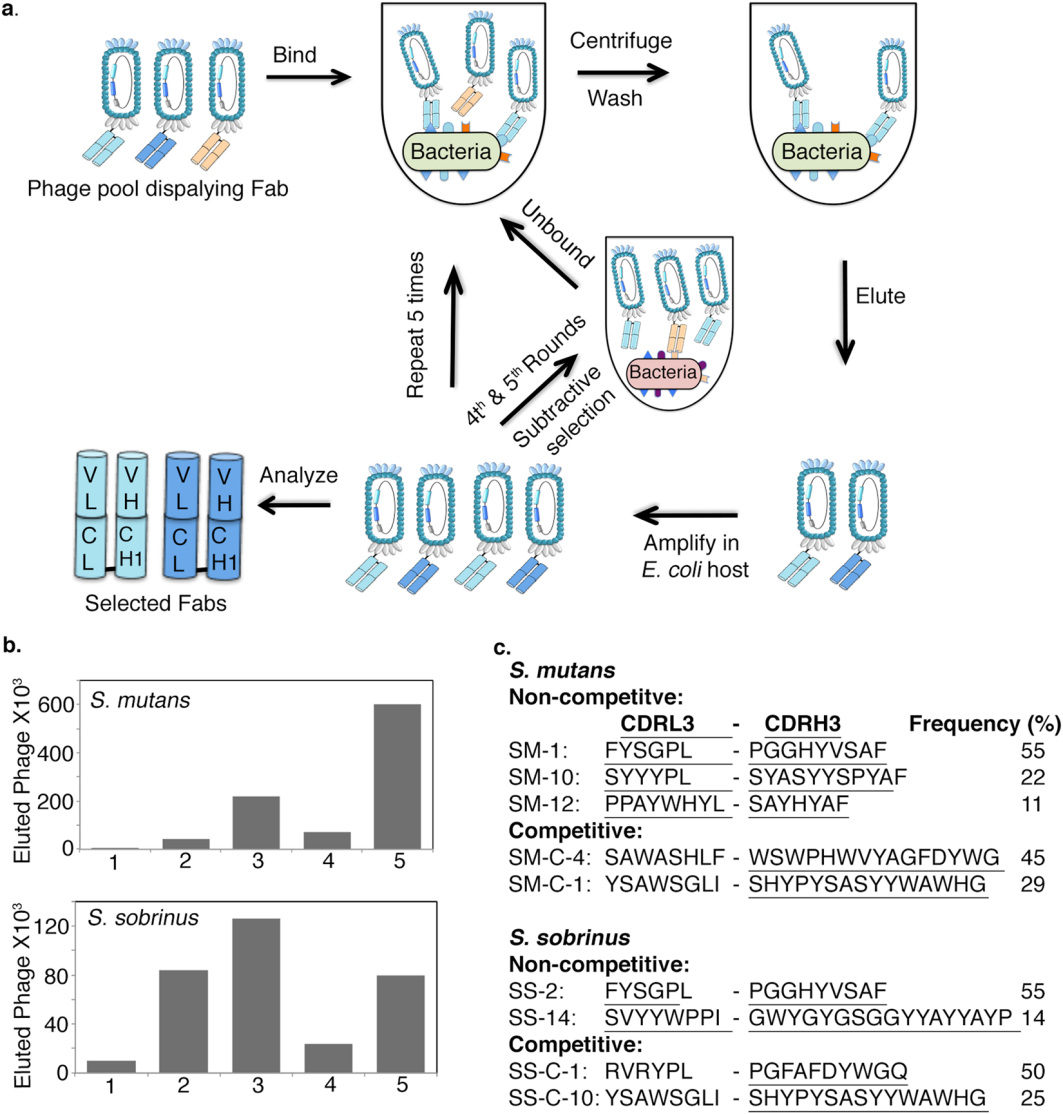
Phage display selection of Fabs against *S*. *mutans* and *S*. *sobrinus*. (**a)** Phage display selection scheme for isolating Fabs against *S*. *mutans* and *S*. *sobrinus*. Five rounds of phage display were performed against *S*. *mutans* or *S*. *sobrinus*. During the fourth and fifth rounds of selection, subtractive panning steps were included. For the *S*. *sobrinus* and *S*. *mutans* selections, members of the Fab-phage library were removed that bound *E*. *coli* and *S*. *mutans* and *S*. *sobrinus*, respectively. (**b)** The frequency of Fab-phage eluted during different rounds of selection against *S*. *mutans* and *S*. *sobrinus*. (**c**) Frequency of the top CDRL3 and CDRH3 sequences of Fabs against *S*. *mutans* and *S*. *sobrinus*.

To generate synthetic human Fabs against *S*. *mutans* and *S*. *sobrinus*, we conducted five rounds of phage display selection using a synthetic Fab phage library^33^ against *S*. *mutans* and *S*. *sobrinus* (Fig. 1a). We observed an increase in phage retention on the bacteria with sequential rounds of selection up to the fifth round (Fig. 1b). During the fourth and fifth rounds of selection, we included subtractive binding steps. For the *S*. *sobrinus* and *S*. *mutans* selections, we removed members of the Fab-phage library that bound *E*. *coli* and *S*. *mutans* and *S*. *sobrinus*, respectively. During selection rounds with subtractive panning, we observed a modest drop in the number of phage retained on *S*. *mutans or S*. *sobrinus* (Fig. 1b). Subsequent rounds of selection resulted in increased phage retention for subtracted phage pools (Fig. 1b). At the end of fifth round, we sequenced 20 phage clones from each selection.

For the *S*. *mutans* selection, sequences present at the highest frequencies were Fabs SM-1, SM-10, and SM-12 (Fig. 1c) and for the *S*. *sobrinus* selection Fabs SS-2 and SS-14 were most frequent sequences (Fig. 1c). The highest frequency Fabs in both selections, SM-1 and SS-2, had identical sequences. We also performed a competitive panning against *S*. *mutans* and *S*. sobrinus using a similar procedure as described above. For competitive panning, we induced *S*. *mutans* and *S*. *sobrinus* to form a biofilm by growing them in presence of sucrose for 4 hours. We then blocked the cell surface of *S*. *mutans* biofilm with Fabs SM-1, SM-10, and SM-12 and the *S*. *sobrinus* biofilm with Fabs SS-2 and SS-14. After 5 rounds of selection, we sequenced 20 clones from the *S*. *mutans* and *S*. *sobrinus* competitive selections. For the *S*. *mutans* selection Fab SM-C1 and SM-C-4 were most frequent Fabs and for the *S*. *sobrinus* selection, Fabs SS-C-1 and SS-C-10 were the most frequent clones. The *S*. *mutans* Fab SM-C-1 and the *S*. *sobrinus* Fab SS-C-10 were the same sequence and were the second most frequent Fab sequence for both *S*. *mutans* and *S*. *sobrinus* competitive selections. Based on their high frequency, we cloned and purified Fab SM-10, SM-12, SM-C-1 (same as SS-C-10), SM-C-4, SS-2 (same as SM-C-1), and SS-C-1 for further characterization. We expressed Fabs in *E*. *coli* and purified them using Protein L chromatography (Fig. S1). Expression yields of these Fabs were in the range of 5–20 mg/L of bacterial culture.

### Fabs binding to *S. mutans* and *S. sobrinus*

**Figure 2:**
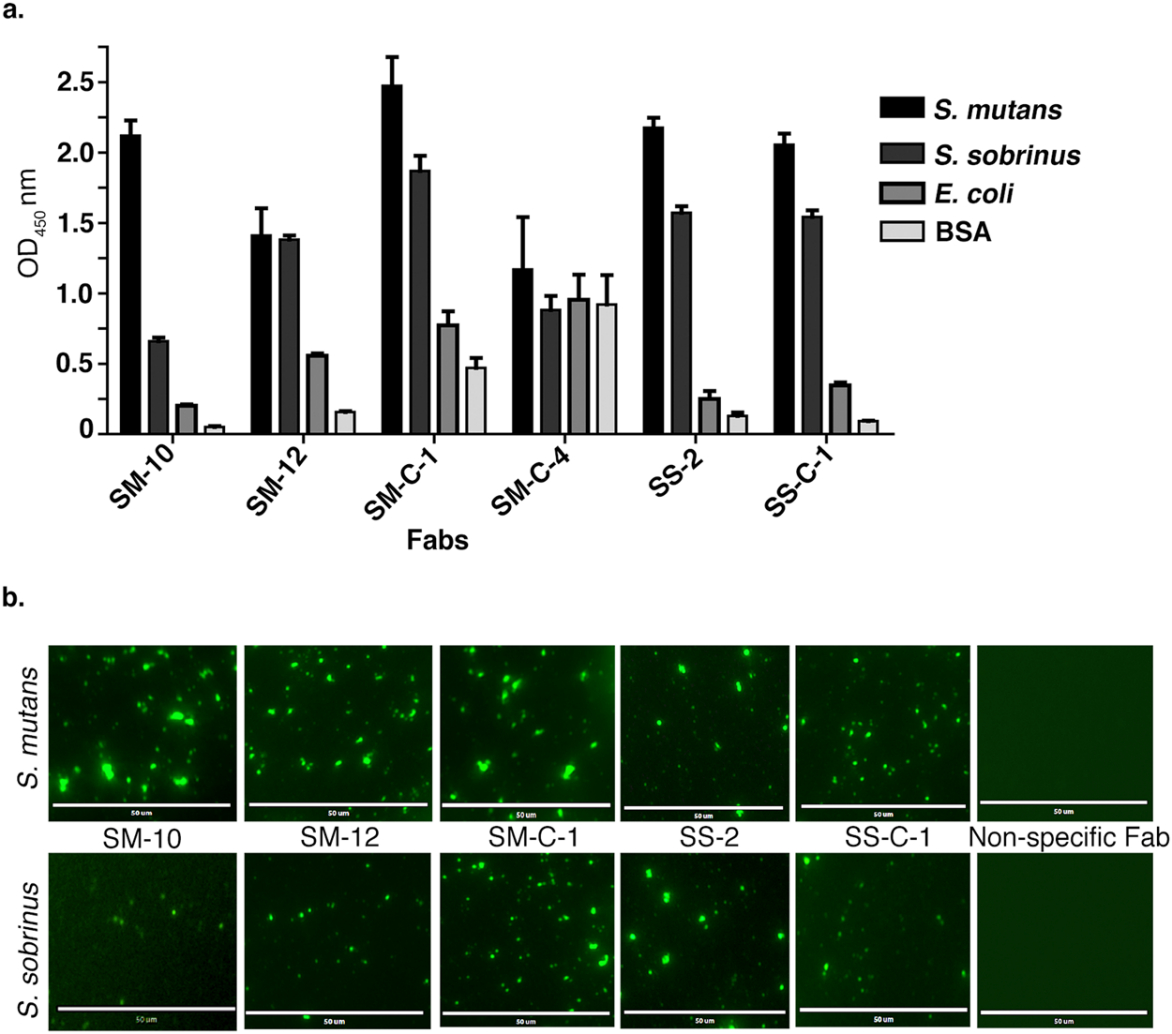
Binding Analyses of anti-*S*. *mutans* and ant-*S*. *sobrinus* Fabs. (**a)** Fab ELISA of Fab SM-10, SM-12, SM-C-1, SM-C-4, SS-2, and SS-C-1 against *S*. *mutans* and *S*. *sobrinus*. *E*. *coli* stain DH10B and bovine serum albumin (BSA) were used as negative controls. Binding of Fabs to bacteria- and BSA-coated microtiter plates was detected using HRP-conjugated anti-His antibody. Error bars represent the standard deviation from three independent experiments. (**b)** Immunofluorescence of selected Fabs against *S*. *mutans* and *S*. *sobrinus*. Non-specific Fab against the human cell surface receptor EphB6 was used as a negative control. Binding of Fabs to bacteria-coated microtiter plates were detected via FITC-conjugated anti-FLAG antibody.

To test the binding selectivity of *S*. *mutans* and *S*. *sobrinus* Fabs, we used ELISA, immunfluorescence, and flow cytometry. We performed the ELISA by adding a fixed concentration of Fabs to *S*. *mutans*, *S*. *sobrinus*, *E*. *coli*, and bovine serum albumin (BSA) immobilized on ELISA plates. Fabs interacted with both *S*. *mutans* and *S*. *sobrinus* and in most cases Fabs bound slightly better to *S*. *mutants* relative to *S*. *sobrinus* (Fig. 2a). SM-C-1 had the highest ELISA signals for both *S*. *mutans* and *S*. *sobrinus* (Fig. 2a). SM-12 Fab bound weaker to both *S*. *mutans* and *S*. *sobrinus*, and SM-C-4 Fab bound non-specifically to *S*. *mutans* and *S*. *sobrinus*, *E*. *coli*, and BSA (Fig. 2a). Except for SM-C-1 and SM-12, Fabs generally bound better to the bacteria that they were selected against, but also showed cross reactivity to *S*. *sobrinus*, indicating that antigens targeted by the Fabs were shared between *S*. *mutans* and *S*. *sobrinus*.

To visualize Fab binding to *S*. *mutans* and *S*. *sobrinus*, we performed immunofluorescence with Fabs SM-10, SM-12, SMC-1, SS-2, and SS-C-1. We coated 10^6^ CFU/mL of bacterial cell suspension on ELISA plates and added 5 μg purified Fabs. Consistent with ELISA results, Fabs bound to both *S*. *mutans* and *S*. *sobrinus* (Fig. 2b).

**Figure 3:**
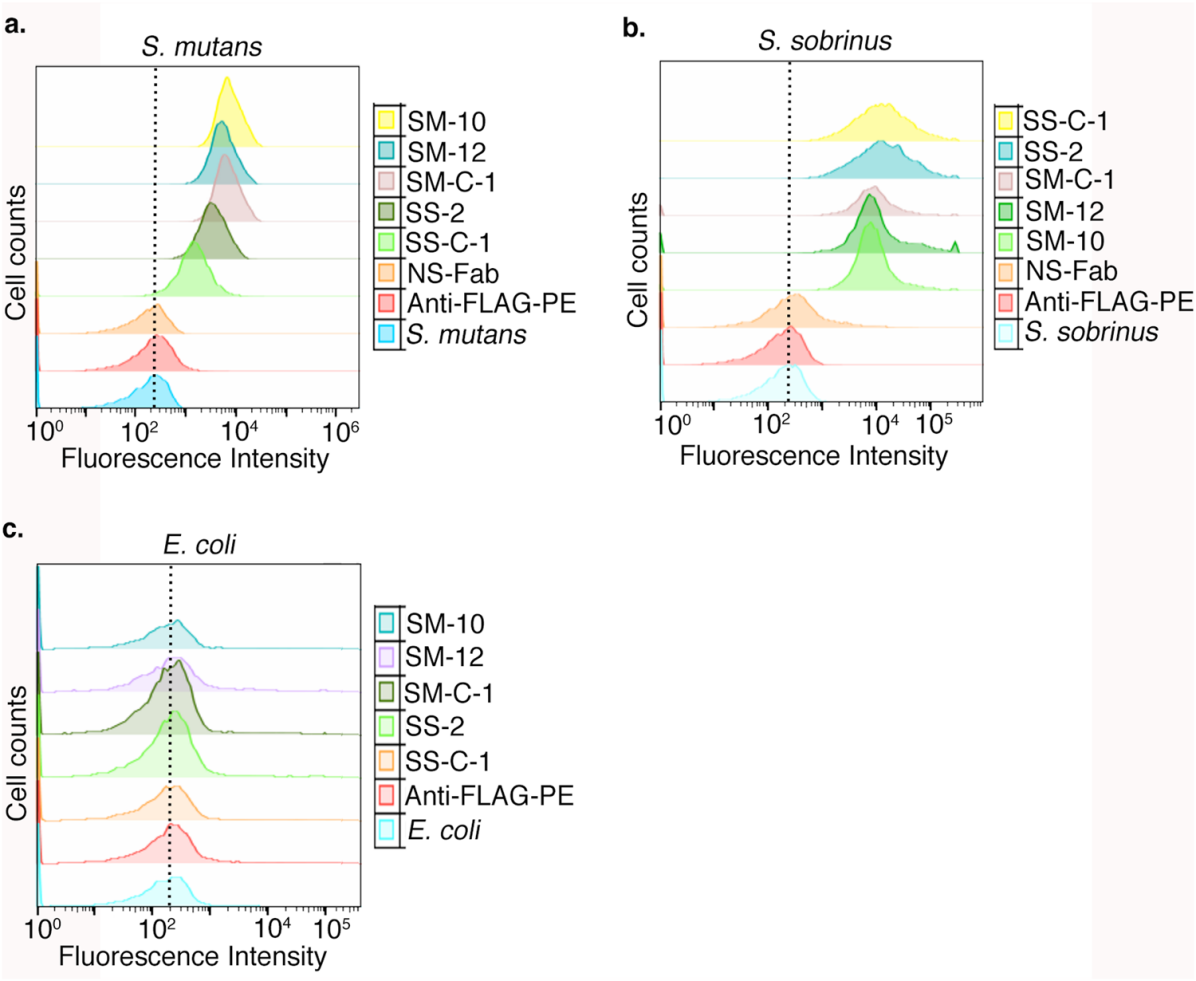
Binding analysis of Fabs to the cell surface of *S*. *mutans*, *S*. *sobrinus*, and *E*. *coli* using flow cytometry. Fabs binding to *S*. *mutans* (**a**). *S*. *sobrinus* (**b**) and *E*. *coli* (**c**) A nonspecific Fab (NS Fab) against human cell surface receptor EphB6 was used a control.

To confirm that Fabs bound to single cell suspensions of *S*. *mutans* and *S*. *sobrinus*, we disrupted filaments of *S*. *mutans* and *S*. *sobrinus* cells by sonication. We added 1 μM Fabs to 10^6^ CFU/tube of suspended bacterial and analyzed binding using flow cytometry with an anti-FLAG R-Phycoerythrin-conjugated secondary antibody. Consistent with ELISA and immunofluorescence results, we observed that SM-10, SM-12, SS-2, and SS-C1 Fabs bound to both *S*. *mutans* and *S*. *sobrinus* with Fabs binding better to the bacteria that they were selected against (Fig. 3a,b). These results were generally consistent with binding results observed in the ELISA. Although the flow cytometry results indicated that the binding of SM-12 to *S*. *mutans* was tighter than SS-2 and SS-C-1, it was the least strongest interaction in the ELISA assay. A nonspecific Fab against human cell surface receptor EphB6 was used as a negative control for Fab binding and it did not bind to *S*. *mutans* and *S*. *sobrinus*. To check the specificity of these Fabs against *S*. *mutans* and *S*. *sobrinus*, a gram-negative *E*. *coli* strain was used as a negative control for bacteria binding and none of the Fabs interacted with *E*. *coli* (Fig. 3c).

In general Fabs bound better to the bacteria that they were selected against, although the *S*. *mutans* Fabs still bound to *S*. *sobrinus* and vice versa, indicating antigens that Fabs targeted were shared between *S*. *mutans* and *S*. *sobrinus*. *S*. *mutans* Fabs SM-1 and SM-C-1 were identical to *S*. *sobrinus* Fabs SS-2 and SS-C-10, respectively, confirming that these Fabs recognized common antigens expressed on *S*. *mutans* and *S*. *sobrinus*. The cross reactivity of Fabs was not unexpected as it has previously been shown that a murine monoclonal antibody against SAI/II protein of *S*. *mutans* recognizes the SAI/II protein of both *S*. *mutans* and *S*. *sobrinus*^26^. Further, a monoclonal antibody against antigen B of *S*. *downei* is specific for both *S*. *sobrinus* and *S*. *downei*^34^.

### *S*. *mutans* and *S*. *sobrinus* Fabs reduced *in vitro* biofilm formation

**Figure 4:**
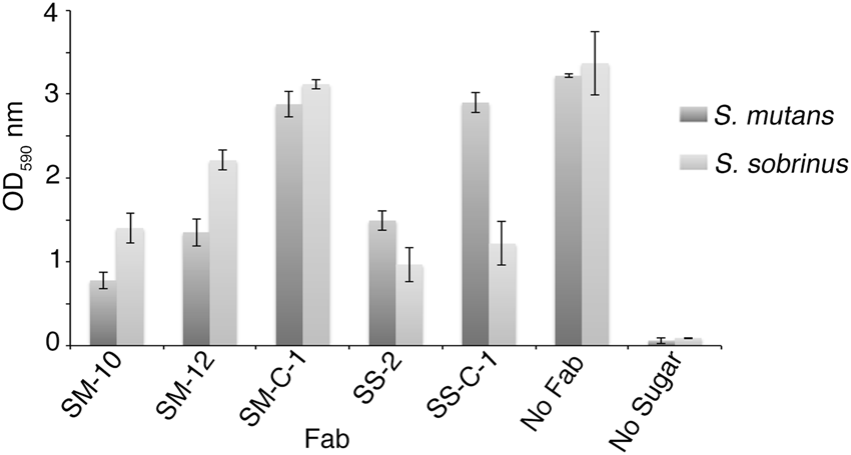
Inhibition of *in vitro* biofilm formation. *In vitro* inhibition of biofilm formation by anti-*S*. *mutans* or anti-*S*. *sobrinus* Fabs. *S*. *mutans* and *S*. *sobrinus* were cultured in brain-heart infusion (BHI) with 1% sucrose to induce biofilm formation in presence of Fabs. Positive control: *S*. *mutans or S*. *sobrinus* on BHI with 1% sugar. Negative control: *S*. *mutans or S*. *sobrinus* on BHI. Error bars indicate standard deviation from three independent experiments.

To assess the effect of *S*. *mutans* and *S*. *sobrinus* Fabs on biofilm formation, we used an *in vitro* biofilm formation assay that measures biofilm formation on a polystyrene plate surface. We added Fabs (3 μM) to cell suspensions of *S*. *mutans* or *S*. *sobrinus* in BHI media with 1% sucrose in a 96 well plate and incubated at 37 °C for 20 hours. *S*. *mutans* or *S*. *sobrinus* cultured in BHI with 1% sucrose were used as positive controls for biofilm formation. *S*. *mutans* or *S*. *sobrinus* cultured in BHI without any sucrose were used as negative controls. Fab SM-10 showed the best blocking of *S*. *mutans* biofilm formation, followed by Fab SM-12 (Fig. 4a). The Fab SS-2 showed the highest inhibition of *S*. *sobrinus* biofilm formation, followed by Fab SS-C-1 (Fig. 4a). None of the Fabs interfered with the normal growth of *S*. *mutans* and *S*. *sobrinus*. SM-10, which blocked *S*. *mutans* biofilm the most, was also the tightest binder to *S*. *mutans*. Fabs SS-2 and SS-C-1 showed the lowest *S*. *mutans* biofilm inhibition, which was consistent with their weak binding properties to *S*. *mutans*. SS-2, which showed the highest *S*. *sobrinus* biofilm reduction, was the strongest binder to *S*. *sobrinus*. In general, we found that Fabs with strong binding properties showed high biofilm inhibition. Previously reports have also shown that polyclonal and monoclonal antibodies to Streptococcal surface antigens inhibit adherence of *S*. *mutans* cells to tooth surfaces^35–37^ and prevent caries formation in non-human primates and in human clinical trials^30^.

### *S*. *mutans* and *S*. *sobrinus* Fabs prevent dental caries formation in rat caries model

**Figure 5:**
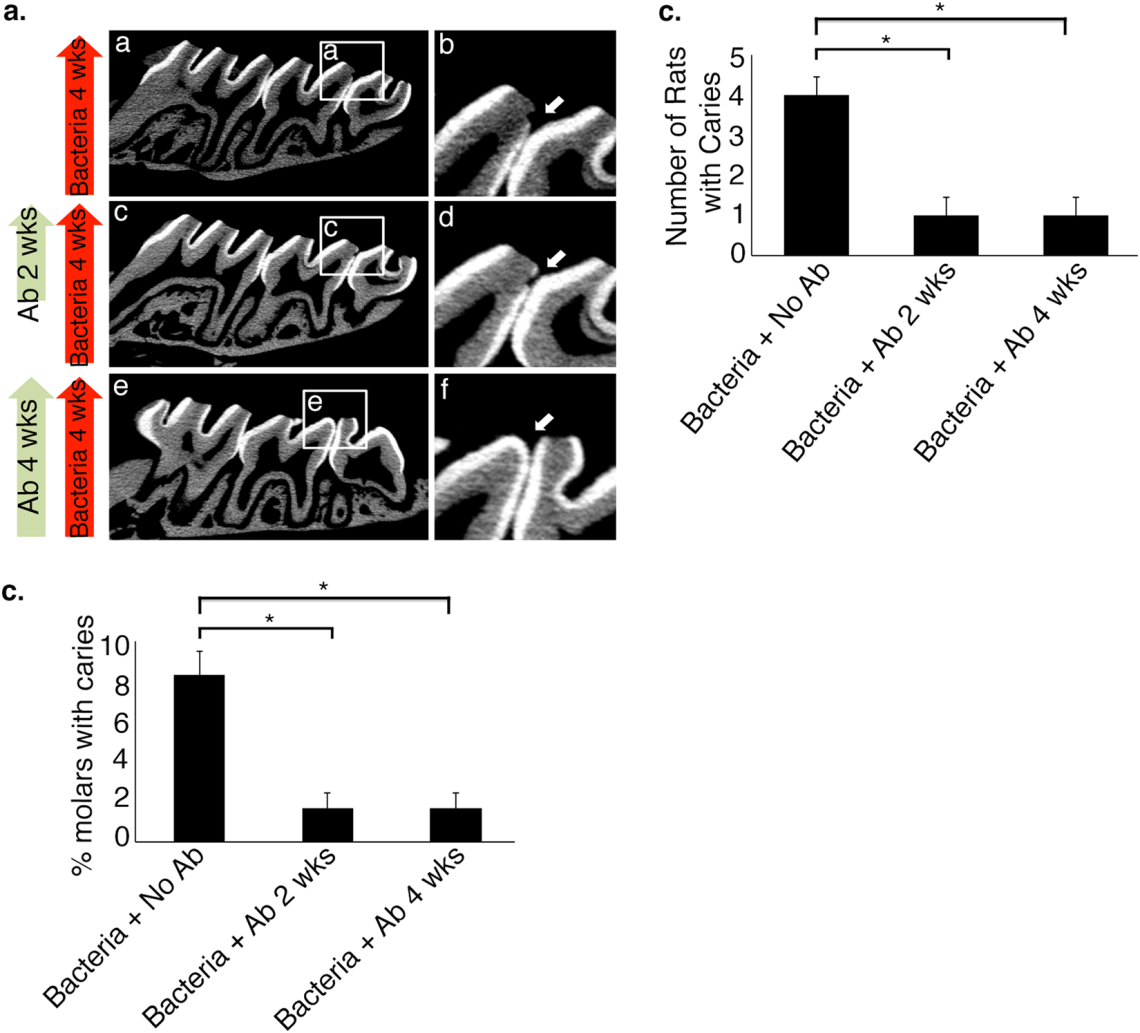
Inhibition of dental caries in rat model. (**a**) Micro-CT analysis of interproximal caries in *S*. *mutans-* and *S*. *sobrinus-*infected rats. For *in vivo* experiments, 15 infection-free rats were randomly divided equally into three groups (group 1, 2 and 3). All groups were infected with *S*. *mutans* and *S*. *sobrinus*. Group 1 did not receive Fabs. Group 2 only received 2 weeks of Fab treatment. Group 3 received 4 weeks of Fab treatment. At the end of the fourth week, rats were sacrificed, mandibles and maxilla were collected and fixed with 70% ethanol for 24 hours and analyzed by micro-CT. (**b**) Number of rats in each group showing caries. Caries were detected and counted by micro-CT. (**c**). Percentage of molars showing caries. Each rat has 12 molars. We checked for caries using micro-CT in a total of 60 molar teeth in each group (5 rats in each group). This experiment was repeated 2 times. Error bars indicate standard deviation. * represents P-values < 0.0001.

For the rat caries model, we used a combination of *S*. *mutans* and *S*. *sobrinus* Fabs that showed the greatest biofilm formation blocking effects *in vitro* (SM-10 and SS-2). Three groups of 20-day old (day 1 after weaning) rats were used for this study. All experimental groups (groups 1, 2 and 3) were infected with *S*. *mutans* and *S*. *sobrinus* at day 1 of the experiment and then two times per week. All groups were also given the cariogenic diet for a total of 4 weeks. For the treatment, we used 50 µg of Fabs and delivered Fabs twice a week in the oral cavity for the duration of the experiment (total of 4 weeks). Group 1 did not receive Fabs (Fig. 5a, a,b). Group 2 only received 2 weeks of Fab treatment (Fig. 5a, c,d). Group 3 received 4 weeks of Fab treatment (Fig. 5a, e,f). At the end of the 4th week, we sacrificed rats and caries were detected by micro-CT. Compared to control group, we observed a significant higher inhibition of caries formation in rats treated with Fabs. Caries were found in four rats out of five in the control group (Fig. 5b), where we observed a total of five caries per sixty molars (Fig. 5c). In contrast, caries were detected only in 1 rat out of five for groups 2 and 3 (Fig. 5b), where we observed one caries per sixty molars (Fig. 5c). There was no difference between 2 weeks and 4 weeks of Fab treatment (local application), suggesting that Fabs show efficacy in this model as early as 2 weeks following the initial application. This experiment was repeated two times with similar results showing an overall 5-fold reduction in total number of dental caries for rats treated with Fabs. This *in vivo* rat model study indicated that combined application of Fabs SM-10 and SS-2 significantly reduced caries formation despite rats receiving a cariogenic diet.

In conclusion, we isolated Fabs that cross-reacted with gram-positive S. mutans and S. sobrinius although at different levels and not with the gram-negative *E*. *coli*, suggesting that they recognize a shared epitope between the gram-positive bacteria. Future work will aim at defining Fab epitopes to determine whether they are specific for mutans streptococci. Epitopes shared by a range of cariogenic bacteria may be show better efficacy in clinical trials, especially for caries when mutans streptococci are present at low levels.”

We showed that synthetic human Fabs could be generated against the cell surface of oral pathogenic bacteria and that oral application of Fabs reduced dental caries in a rat caries model. This strategy allows Fab production in large quantities in microbial culture systems, which would be more suitable to scale up for the routine control of dental caries. Secretory IgEs are generally more preferable for oral applications as they are most abundant in saliva, resistant to proteolysis, and have higher retention time in oral cavity compare to IgGs or Fabs^38^. Since synthetic Fabs are made using recombinant DNA technology, our system allows Fabs to be engineered easily to make human secretory IgE or IgG. This would prevent adverse immune responses seen with nonhuman antibodies. Passive immunization using synthetic Fabs is much safer and easier to administer locally compared with approaches using active vaccination. In the future, this strategy might lead towards an inexpensive and convenient general treatment for dental caries.

## Methods

### Bacterial strains and culture conditions

*S*. *mutans* (ATCC700610) and *S*. *sobrinus* (ATCC27351) were cultured on brain heart infusion (BHI) agar (Difco) for 24–48 h at 37 °C and 5% CO_2_. Single colonies were isolated and used to inoculate 3 mL of BHI and cultured overnight at 37 °C with 5% CO_2_. For phage display, 10 mL of BHI was inoculated with 0.5 mL of an overnight culture and grown at 37 °C with 5% CO_2_ to an OD_600_ of 1.0. For biofilm- induced competitive panning, 10 mL of BHI with 1% sucrose was inoculated with 0.5 mL of an overnight culture and grown at 37 °C with 5% CO_2_ for 3–4 hours. For biofilm assay, overnight cultures of *S*. *mutans* and *S*. *sobrinus* were prepared to a final concentration of 10^6^ CFU/mL and grown in BHI with 1% sucrose for 20 hrs at 37 °C in a 5% CO_2_ aerobic atmosphere.

### Selection protocol

The selection protocol is outlined in Fig. 1a. Target antigens for panning were live *Streptococcus mutans* and *Streptococcus sobrinus cells*. The subtraction panning was performed using *E*. *coli* (DH10B) and *S*. *mutans* (in case of *S*. *sobrinus*) or *S*. *sobrinus* (in case of *S*. *mutans*). For the selection, 10 mL of BHI was inoculated with overnight cultures of *S*. *mutans* or *S*. *sobrinus* and cultured until they reached an OD_600_ of 1. Bacteria were pelleted by centrifugation and used for selections. For sucrose-induced biofilm competitive selections, 10 mL of BHI supplemented with 1% sucrose was inoculated with overnight cultures of *S*. *mutans* or *S*. *sobrinus* and cultured at 37 °C with 5% CO_2_ for 4 hours. Bacteria were centrifuged and cell pellets were collected for selections. Approximately 10^9^ bacterial cells were washed twice with phosphate-buffered saline (PBS), re-suspended in 1 ml of PBS containing 1% BSA, and incubated in a 1.5 mL micro-centrifuge tube on a rotator for 1 hr at room temperature. This and subsequent incubations were carried out using micro-centrifuge tubes that had been blocked with 1% BSA in PBS for 1 hr at room temperature. After 1 hr, 10^13^ phage particles containing antibody fragments (Fabs) were added to the tube containing 10^9^ cells in 500 uL PBS-1% BSA and rotated for an additional 1 hr at room temperature to allow phages to bind to the bacteria. After incubation, selection tubes were washed eight times with PBS containing 0.05% Tween-20 (PBT) to remove unbound phages. During final wash, re-suspended bacteria cells were moved to a fresh tube that was pre-blocked with 1% BSA in PBS. To elute bound phages, the cell pellet was re-suspended in 500 uL of 100 mM HCl and rotated at room temperature for 5 min. The tube was centrifuged for 2 min at 10,000 RPM and the supernatant containing eluted phages was transferred to a new micro-centrifuge tube and neutralized with 1 M Tris–HCl pH 8.0. Eluted phages were used to infect NEBrαF’ *E*. *coli* cells (NEB). Phage were either amplified for use in the next round of selection or plated on LB media containing carbenicillin and kanamycin to isolate *E*. *coli* harboring individual phagemids. Subtractive panning was performed at rounds 4 and 5. During subtractive panning, phage pools amplified from round three were first incubated with *E*. *coli* and then the unbound phage pool was further incubated with *S*. *mutans* (in case of *S*. *sobrinus*) or *S*. *sobrinus* (in case of *S*. *mutans*). During sucrose-induced biofilm competitive selections, suspended bacteria were first incubated with Fabs that were isolated from non-biofilm selections and then phage particles were added to the bacteria.

### DNA sequencing

At the end of the fifth round of selection, phage infected NEBrαF‘ *E*. *coli* cells were plated on LB media containing antibiotic carbenicillin and kanamycin to isolate single colonies. Colony PCR was performed to confirm the correct size of the Fab. For Sanger sequencing, phagemids from twenty *E*. *coli* colonies after fifth round of panning were isolated. Phagemids were sequenced on 3500 Genetic Analyzer (Thermo Fisher Scientific) using TGS163 and TGS164 primers (Table S1).

### Fab Cloning, Expression, and Purification

Selected Fabs were PCR-amplified using TGS157 and TGS160 primers (Table S1) from phagemids. The PCR-amplified Fab amplicon was cloned into *SacI*- and *XhoI*-digested pCW-LIC Fab expression vector (Addgene) using Gibson assembly^39^. Each Fab contained the light chain signaling sequence, light chain (VL-CL), FLAG tag, heavy chain signaling sequence, heavy chain (VH-CH1), GGS linker, and C-terminal His_6_ tag in the order listed. Fab expression plasmids were transformed into BL21 (DE3) competent *E*. *coli* cells (NEB) and plated on 2YT media containing carbenicillin. Single *E*. *coli* colonies were cultured in overnight expression Instant TB media (Novagen) for 20–24 hrs^40^. Cells were pelleted and suspended in Protein L Binding buffer (Sodium Phosphate 20 mM, 0.15 M NaCl, pH 8.0). Cells were disrupted at 35 Kpsi using the cell disruptor (Constant System LTD. USA). Cell debris was removed by centrifuging at 12,000 RPM for 20 min. Supernatant was collected and filtered through a 0.45-micron membrane filter (Minisart, Sartorius stedim). Fabs were purified on a GE Healthcare AKTA FPLC system using a HiTrap Protein L column (GE Heathcare). Fabs were eluted from the Protein L column using IgG elution buffer (Fisher Scientific) and neutralized with neutralization buffer (1 M Tris-HCl pH 9.0). Purified Fabs were dialyzed overnight in PBS and concentrated using 10 K MWCO filter (Millipore). Fabs were filter sterilized and stored at −20 °C. Fabs were quantified using the Bicinchonic acid assay (Pierce™ BCA Protein Assay Kit, Thermo Scientific) following manufacturer’s instructions.

### Enzyme linked immunosorbent assay (ELISA)

To test the binding of phage display generated Fabs, 96-well Maxisorp ELISA plates (Nunc) were coated with bacterial cell suspension containing 10^7^ cells per well in PBS for 1 hr at 37 °C followed by overnight at 4 °C. Plates were washed three times with PBS and blocked with 2% milk powder in PBS for 2 hrs at room temperature. Purified Fabs (5 μg per well) were incubated with cells for 2 hrs at room temperature. After incubation with Fabs, the plate was washed three times with PBST (PBS containing 0.01% Tween 20) followed by an additional three washes with PBS. Cell bound Fabs were detected using anti-His horseradish peroxidase-conjugate (Amersham Biosciences, Piscataway, NJ, USA). ELISAs were developed with 3,3′,5,5′-tetramethylbenzidine (TMB) (Sigma). Reactions were stopped by the addition of H_2_SO_4_ after 20 min and readings taken at OD_450nm_ using plate reader (Spectra Max, Molecular Devices).

### Immunofluorescence

Bacterial cell suspensions at a concentration of 10^6^ CFU/mL were coated on 96-well plate and blocked with 2% milk powder in PBS for 1 hr at room temperature. Purified Fabs (5 μg per well) were incubated with bacteria cells for 1 hr at room temperature. Following three washes with 1× PBS, FITC conjugated Anti-FLAG antibody was added at 1:5000 dilution to each well and incubated for 1 hr at room temperature. Bacteria were washed three times in PBST followed by three additional washes with PBS. Fluorescence images were taken using fluorescence microscope (EVOS, Advance Microscopy Group).

### Flow cytometry

Bacteria growing on BHI were centrifuged and washed once with PBS. To break filaments of *S*. *mutans* and *S*. *sobrinus*, *bacteria* cells were re-suspended in 1 mL of ice-cold PBS and sonicated using a microtip probe sonicator for 20 secs at 10% power with a setting of a 0.5 second impulse, followed by a 0.5 sec break. For flow cytometry analyses, bacterial cell suspensions at a concentration of 10^6^ CFU/tube were incubated with different Fabs (1 μM) at room temperature for 60 min. Bacteria cells were washed three times with PBS, pH 7.4. Anti-FLAG® R-Phycoerythrin Conjugate (ProZyme) was added at 1:3000 dilution to each tube and incubated for 30 min at room temperature. Bacteria cells were washed three times in PBST followed by three additional washes with PBS and re-suspended in 500 μL of PBS. Bacteria cell fluorescence was monitored using a flow cytometer (MACS Quant, Miltenyi Biotec) with 561 nm excitation and 586–541 nm emission wavelengths. Flow cytometry data were analyzed using Flowjo (Tree Star).

### *In vitro* biofilm assay

The effect of different Fabs on *S*. *mutans* and *S*. *sobrinus* biofilm formation were examined using the microdilution method^41^. Briefly, overnight culture of *S*. *mutans* and *S*. *sobrinus* were prepared to a final concentration of 10^6^ CFU/mL and grown in BHI for 24 hrs at 37 °C in a 5% CO_2_ aerobic atmosphere with the addition of 1% sucrose for biofilm development. Different Fabs were added to the culture at 0 hour at a final concentration of 3 μM. After 24 hours, before biofilm quantification, the culture growth was measured at OD_600nm_ using a plate reader. The cell suspension was carefully removed and each well was washed three times with 1× PBS. The remaining attached cells were fixed with 96% ethanol for 15 min and then plates were dried. Wells were stained with 120 μL of 0.1% crystal violet (Fisher) for 20 minutes. The crystal violet stain was aspirated and wells were washed three times with 150 μL of PBS. Wells were air-dried and retained crystal violet was solubilized with 150 μL of 30% (v/v) glacial acetic acid (Fisher). The plate was incubated at room temperature for 20 min with shaking. The biofilm formation was quantified by measuring the OD_590nm_ using a plate reader.

### *In vivo* rat caries model

The animal experiment protocol was reviewed and approved by the Ethical Committee on Animal Research at the University of Michigan. Fifteen days old Sprague-Dawley rats were purchased from Charles River (Wilmington, MA). Rats then were screened for infection with *S*. *mutans* and *S*. *sobrinus*. Fifteen infection-free rats were given *S*. *mutans* and *S*. *sobrinus* (kindly proved by Dr. Christopher Fenno, School of Dentistry, University of Michigan) twice a week for 4 weeks starting at day 21 after birth. All rats were fed high cariogenic diet 2000 (Envigo, Madison, WI) and 5% sucrose water *ad libitum*^42^. Rats were randomly divided into three groups. Each group had 5 rats. All groups received the bacteria mixture applied directly on their teeth with a brush twice a week for total 4 weeks. Rats in the first group were treated with a mixture of Fabs (SM-10 and SS-2) applied directly on teeth surface using a brush starting at day 1 of the experiment. The second group was treated with the same Fabs mixture twice a week starting from the 3rd week for a total of two weeks; the last group was the vehicle control group; rats in this group were treated with PBS twice a week for 4 weeks. Upon completion of the 4 weeks the rats were sacrificed under anesthesia with a ketamine/xylazine mixture and mandibles and maxilla collected, fixed with 70% ETOH for 24 hours and analyzed by micro-CT.

### Micro-CT

The dissected samples were scanned with micro-computed tomography (SkyScan 1272 Micro-CT at 60 kV, with 6.0 um pixel size). The scanned images were reconstructed with Nrecon (Bruker, Manning Park, MA) and DataViewer (Bruker).

### Availability of data and materials

The datasets used and/or analyzed during the current study available from the corresponding author on reasonable request.

## Electronic supplementary material

Supplementary Information
