# Author Correction: Synthetic antigen-binding fragments (Fabs) against *S. mutans* and *S. sobrinus* inhibit caries formation

**DOI:** 10.1038/s41598-020-71086-8

**Published:** 2020-09-09

**Authors:** Md. Kausar Alam, Li Zheng, Ruirui Liu, Silvana Papagerakis, Petros Papagerakis, C. Ronald Geyer

**Affiliations:** 1 Department of Pathology and Laboratory Medicine, College of Medicine, University of Saskatchewan, Room 2841, Royal University Hospital, 103 Hospital Drive, Saskatoon, S7N0W8 Canada; 2 Department of Orthodontics and Pediatric Dentistry, School of Dentistry, University of Michigan, Ann Arbor, 1011N University, Ann Arbor, 48109 USA; 3 Key Laboratory of Shaanxi Province for Craniofacial Precision Medicine Research, Xi’an Jiaotong University, Xi’an, 710004 People’s Republic of China; 4 Department of Surgery, College of Medicine, Health Sciences Center, 107 Wiggins Road, Saskatoon, SK S7N 5E5 Canada; 5 College of Dentistry, Health Sciences Center, 107 Wiggins Road, Saskatoon, SK S7N 5E5 Canada

Correction to: *Scientific Reports* 10.1038/s41598-018-28240-0, published online 05 July 2018

The original version of this Article contained a typographical error in Affiliation 2. The correct affiliation is listed below:

Department of Orthodontics and Pediatric Dentistry, School of Dentistry, University of Michigan, Ann Arbor, 1011N University, Ann Arbor, 48109, USA

Additionally, the original version of this Article omitted an affiliation for Ruirui Liu. The missing affiliation is listed below:

Key Laboratory of Shaanxi Province for Craniofacial Precision Medicine Research, Xi'an Jiaotong University, Xi'an, 710004, People’s Republic of China

These errors have now been corrected in the PDF and HTML of the original Article, and in the accompanying supplementary material.

