# Supplementary material for: Synthetic antigen-binding fragments (Fabs) against S. mutans and S. sobrinus inhibit caries formation
Source: Sci Rep. 2018 Jul 5;8:10173. doi: 10.1038/s41598-018-28240-0 (PMC6033933; doi:10.1038/s41598-018-28240-0)
Supplement: Supplementary file 1 — Supplementary Information [file 41598_2018_28240_MOESM1_ESM.docx]

**Synthetic antigen-binding fragments (Fabs) against *S. mutans* and *S. sobrinus* inhibit caries formation**

Md. Kausar Alam^1^, Li Zheng^2^, Ruirui Liu^2, 3^, Silvana Papagerakis^4^, Petros Papagerakis^*2, 5^, C. Ronald Geyer^*1^

**Author’s affiliation:**

**^1^** Department of Pathology and Laboratory Medicine, College of Medicine, University of Saskatchewan, Room 2841, Royal University Hospital, 103 Hospital Drive, Saskatoon, S7N0W8, Canada

^2^ Department of Orthodontics and Pediatric Dentistry, School of Dentistry, University of Michigan, Ann Arbor, 1011 N University, Ann Arbor, 48109, USA

^3^ Key Laboratory of Shaanxi Province for Craniofacial Precision Medicine Research, Xi'an Jiaotong University, Xi'an, 710004, P. R. China

^4^ Department of Surgery, College of Medicine, Health Sciences Center, 107 Wiggins Road, Saskatoon, SK, S7N 5E5, Canada

^5^ College of Dentistry, Health Sciences Center, 107 Wiggins Road, Saskatoon, SK, S7N 5E5, Canada

^*^ Author for correspondence:

C. Ronald Geyer Tel: 306-966-2040; email: [ron.geyer@usask.ca](mailto:ron.geyer@usask.ca)

Petros Papagerakis, Tel: 306-966-5116; email: [petros.papagerakis@usask.ca](mailto:petros.papagerakis@usask.ca)


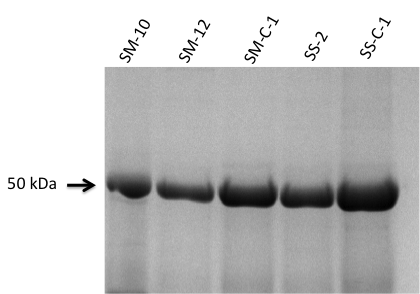


**Figure S1: Non-reducing SDS-PAGE analysis of purified *S. mutans* and *S. sobrinus* Fabs.** Fabs were expressed in *E. coli* and purified using Protein L chromatography. All Fabs have molecular weight of approximately 50 kDa. The light chain of all Fabs has the organization of CL-VL-FLAG tag. The heavy chain of all Fabs has the organization of CH1-VH-His_6_ tag.

**Table S1: List of primers used in this study**

| **List of primers** | |
| --- | --- |
| **Name** | **Sequence** |
| TGS157 | 5- TCCAGATGACCCAGTCCCCGAGCTCCCTG-3 |
| TGS 160 | 5- CAA ATC TTG TGA CAA AAC TCA CAC GGG TGG TTC GCA CCA CCA CCA CCA CCA CTG AG -3 |
| TGS163 | 5- GGAAACGGATCAGCTTACTCC -3 |
| TGS164 | 5- CTAAGAAACCATTATTATCATGAC-3 |
|  |  |
|  |  |
|  |  |
|  |  |
